# Barriers and facilitators of seasonal influenza vaccinations in German and Chinese health care workers - a comparative cross-sectional study

**DOI:** 10.1186/s12889-026-29260-6

**Published:** 2026-09-01

**Authors:** Linda Sanftenberg, Yiqing Weng, Hongmei Wang, Robert Philipp Kosilek, Jochen Gensichen

**Affiliations:** 1 Institute of General Practice and Family Medicine, LMU University Hospital, Nußbaumstr. 5, Munich, 80336 Germany; 2 Department of Social Medicine, School of Public Health, Zhejiang University, 866 Yuhang Tang Rd, Hangzhou, 310058 China

**Keywords:** Vaccination readiness, Health care workers, Seasonal influenza, China, Germany, Implementation science

## Abstract

**Background:**

Influenza vaccination coverage among health care workers (HCWs) remains insufficient in different health care systems. To develop and implement effective interventions, a structured comparison of the psychological as well as context-driven determinants of vaccination readiness among HCWs from two different health care systems can be extremely helpful. The aim of this study was to elaborate barriers and facilitators on vaccination readiness among HCWs within the German and Chinese health care system.

**Methods:**

Two independent online surveys among HCWs on influenza vaccination have been performed 2024 in Germany and 2025 in China. Differences in vaccination behavior were tested for age, occupation, and main reasons for refusing the seasonal influenza vaccination were asked. To identify the main drivers in both health care systems, these findings have been compared according to consolidated framework for implementation research (CFIR).

**Results:**

Altogether, *n* = 8.351 German HCWs (*n* = 72 German hospitals), as well as *n* = 689 Chinese HCWs (*n* = 9 Chinese hospitals) had been surveyed. Concerning vaccination behavior, 80.9% of the surveyed German physicians and 28.8% of the Chinese physicians stated to be vaccinated. Among the surveyed nurses, 48.7% German and 32.2.% Chinese nurses had received the influenza vaccination. The main psychological determinants for vaccination readiness among German HCWs and Chinese HCWs were “confidence” (German HCWs mean 3.9; Chinese HCWs 4.4) and “collective responsibility” (German HCWs mean 3.8; Chinese HCWs 4.5). The psychological antecedent “calculation” was weighted differently in both populations (German HCWs mean 3.5; Chinese HCWs mean 4.4). Main reasons to refuse the vaccination were located in various implementation domains, as most of the German HCWs pronounced individual aspects (e.g. negative risk-benefit assessment), whereas Chinese HCWs emphasized barriers within the inner setting of their medical facilities (e.g. time restricitions).

**Discussion:**

HCWs influence influenza vaccine uptake as both providers and recipients. Their decisions are shaped by confidence and collective responsibility, as well as different implementation factors. Nurses and physicians show contrasting uptake patterns in Germany and China, influenced by training differences and healthcare system structures. Effective interventions include free of charge vaccination policies, education, and organizational measures.

**Trial registration:**

Not applicable.

## Introduction

Globally, influenza causes an estimated 1 billion infections each year, including 3–5 million severe cases, and results in approximately 290,000–650,000 influenza-related respiratory deaths [1]. In medical facilities with a high proportion of vulnerable individuals and a high outbreak potential, limiting the spread of infectious diseases is particularly important [2]. Vaccination is recognized worldwide as the most successful and cost-effective intervention to reduce the burden of infectious disease. It plays a crucial role in preventing and controlling infections and in safeguarding public health and safety [3]. As a high-risk group, healthcare workers (HCWs) are recommended to receive annual influenza vaccination. The seasonal influenza vaccination, protects staff from the disease, reduces the spread of the virus within the hospital and can reduce the transmission of the influenza virus in facilities with vulnerable patients [4]. However, past surveys have indicated significant vaccination gaps among HCWs for several years. The average vaccination rate among hospital staff had never exceeded 60% in the last years [5, 6].

There is a number of psychological, contextual, sociodemographic, and pragmatic barriers to vaccination uptake against seasonal influneza among adults [7]. A lack of trust in the safety and effectiveness of vaccines, organizational hurdles, the active search for and evaluation of information, and the perceived risk of the infectious diseases to be prevented are cited to varying degrees as barriers to influenza vaccination [8]. In addition to incomplete knowledge about potential side effects, a misconception about susceptibility to certain diseases and their impact on the health are also important reasons for low uptake [9]. Reduced social responsibility and a lack of understanding the need for community protection also influence individual motivation for recommending the vaccination as well as the personal uptake [10].

Comparing German HCWs with Chinese HCWs allows for examining the influence of cultural and environmental backgrounds on vaccination decisions. The rationale of comparing these two countries has to do with the differing sociocultural, political, and public health contexts that may influence attitudes towards vaccination. Germany and China vary significantly in terms of healthcare infrastructure, vaccine policies, and social values [11].

Many interventions that have proven effective in clinical trials cannot be readily transferred to everyday healthcare practice [12]. Various barriers and facilitators, both on the individual behavioural as well as the contextual level, can hinder successful implementation [13]. Implementation science encompasses both general theory building and the development of practical approaches for successful implementation; both researchers and practitioners use and benefit from these models [14]. The aim of the present analyses was to elaborate barriers and facilitators on vaccination readiness among HCWs within the German and Chinese health care system.

## Material and methods

### Data collection

#### Study population of the German survey

The data collection took place from 22.04.24 to 02.06.2024, via an online survey on the VOXCO platform of the Robert Koch-Institute (RKI). At the start of the survey, the link to the online questionnaire was sent to the contact persons within the participating hospitals, who then forwarded it to staff via email and the hospital intranet. Participation in the survey was voluntary. Written informed consent was obtained from all study participants prior data collection. As an incentive to participate in the study, respondents could enter a prize draw after completing the questionnaire.

#### Study population of the Chinese survey

The data collection was conducted between 04.07.2025 to 26.10.2025, across different community health centers in Zhejiang Province, China. Eligible participants were HCWs employed in a variety of professional roles and departments within these centers. Recruitment was voluntary and based on convenience sampling. Data collection was performed using an online questionnaire platform. Written informed consent was obtained from all study participants prior data collection. As an incentive to participate in the study, respondents could enter a prize draw (10–20 CNY bonus) after completing the questionnaire.

#### Questionnaire of the German survey

Study participants provided demographic and occupational information, including age, sex, their professional role (physician, nurse, medical technician, laboratory personnel, administration, or other) and the respective department of employment (frontline patient care, support and diagnostics, administration and public health, or other). Influenza vaccination status was self-reported for the 2023/2024 season. Participants also indicated whether their workplace offered free influenza vaccination services. The questionnaire was designed on the basis of literature research and in consultation with vaccination experts. Moreover, the German questionnaire included eleven potential reasons against receiving the influenza vaccine (multiple answers possible). Psychological determinants of vaccination readiness were measured using the German version of the 5 C scale. The German version of the 5 C scale was rated on 5-point Likert scale ranging from 1 (strongly disagree) to 7 (strongly agree) [8].

#### Questionnaire of the Chinese survey

Study participants provided demographic and occupational information, including age, sex, their professional role (physician, nurse, medical technician, laboratory personnel, administration, or other) and the respective department of employment (frontline patient care, support and diagnostics, administration and public health, or other). Influenza vaccination status was self-reported for the 2024/2025 season. Participants also indicated whether their workplace offered free influenza vaccination services, and if they are willing to recommend this vaccination to their patients (yes/no). The Chinese questionnaire referred to nine potential reasons against the reception of the annual influenza vaccination (selecting and ranging the three main reasons). Psychological determinants of vaccination readiness were measured using the Chinese version of the 5 C questionnaire [15, 16]. The Chinese version of the 5 C scale was rated on a 7-point Likert scale ranging from 1 (strongly disagree) to 7 (strongly agree). Domain scores were calculated as the mean within each domain, resulting in continuous scores from 1 to 7, with higher scores reflecting a greater degree of the corresponding attitude.

#### Consolidated Framework for Implementation Research

To compare barriers and facilitators on vaccination readiness among HCWs within the German and Chinese health care system on a contextual level, we have applied the Consolidated Framework for Implementation Research (CFIR). The CFIR provides a conceptual model of implementation drivers across five domains, namely intervention characteristics, inner setting, outer setting, characteristics of the individual and process of implementation [17].

### Data analysis

Statistical analyses were conducted using IBM SPSS 25.0 (IBMCorp., Armonk, N.Y., USA) and Stata 19.0 (StataCorp, College Station, TX, USA). Data was complete for all variables included in the analysis. Descriptive statistics and regression analysis were calculated for all variables as appropriate for the respective type and distribution. We used complete cases analysis since < 2.5% of the data were missing. Missing data were not replaced or imputed. We decided to analyze the different data sets separately, because of the different health care systems and terms data collection. The dichotomous outcome variable was whether participants were vaccinated against influenza in the respective season or not. We included sociodemographic characteristics (age, gender, occupation, department), access to the vaccine (offered at workplace or not), as independent variables (all categorical). We have reported associations with *p* < .05 as statistically significant. Domain scores of the 5 C scale were calculated as the mean within each domain, resulting in continuous scores from 1 to 5 (German survey), respectively from 1 to 7 (Chinese survey). To compare the results of both surveys, the results of the Chinese questionnaire were aligned using linear transformation (x5 = 6(x7 − 1)⋅4 + 1).

## Results

### Study population

In total, 72 German hospitals with *n* = 8.351 Geman HCWs and nine Chinese community health centers with *n* = 689 Chinese HCWs participated in the respective survey. The mean age of the study participants was 41 years, and 23.9% of the study participants were male. The nursing staff (30.9%) was the largest occupational group in the sample, followed by administrative and public health staff (21.3%). Almost half of the study participants worked in frontline patient care (47.9%). Around 90.5% of all participants reported that the influenza vaccine was offered at their workplace. However, just 58% of the surveyed German HCWs and 31% of the Chinese HCWs had received an influenza vaccination. Further details on the study population in all seasons can be found in Table 1.

**Table 1:** Baseline characteristics of the study participants

Sample characteristic^1^	German HCWs(n=8.351)	Chinese HCWs(n=689)
Sociodemographic data		
Male gender, N (%)	2.066 (24.7%)	159 (23.1%)
Age group in years, N (%)		
18-30	1.443 (17.3%)	196 (28.4%)
31-40	1.947 (23.3%)	225 (32.7%)
41-50	1.923 (23.0%)	198 (28.7%)
51-60	2.283 (27.3%)	69 (10.0%)
>60	766 (9.2%)	1 (0.1%)
Profession		
Physician	1.356 (16.2%)	267 (38.8%)
Nurse	2.569 (30.8%)	230 (33.4%)
Administrative	1.841 (22.0%)	87 (12.6%)
Medical technician	433 (5.2%)	105 (15.2%)
Others	2.079 (24.9%)	0 (0%)
Department		
Frontline patient care	4.056 (48.6%)	283 (41.1%)
Support and diagnostics	1.860 (22.3%)	163 (23.7%)
Administration & Public Health	2.446 (29.3%)	243 (35.3%)
Yes	7.686 (92.0%)	613 (89.0%)
No	79 (0.9%)	34 (4.9%)
Unknown	596 (7.1%)	42 (6.1%)
Influenza vaccination rates according to profession		
Physician	80.9%	28.8%
Nurse	48.7%	32.2%
Administrative	47.4%	32.2%
Medical technician	59.2%	29.5%
Others	62.4%	0%
Influenza vaccination rates according to age		
Age group in years		
18-30	35.8%	24.5%
31-40	51.9%	29.3%
41-50	59.0%	33.3%
51-60	68.5%	43.5%
>60	81.2%	100.0%

^1^Reported as n (%) unless otherwise specified

### Potential reasons for refusing vaccinations against seasonal influenza

**Fig. 1:**
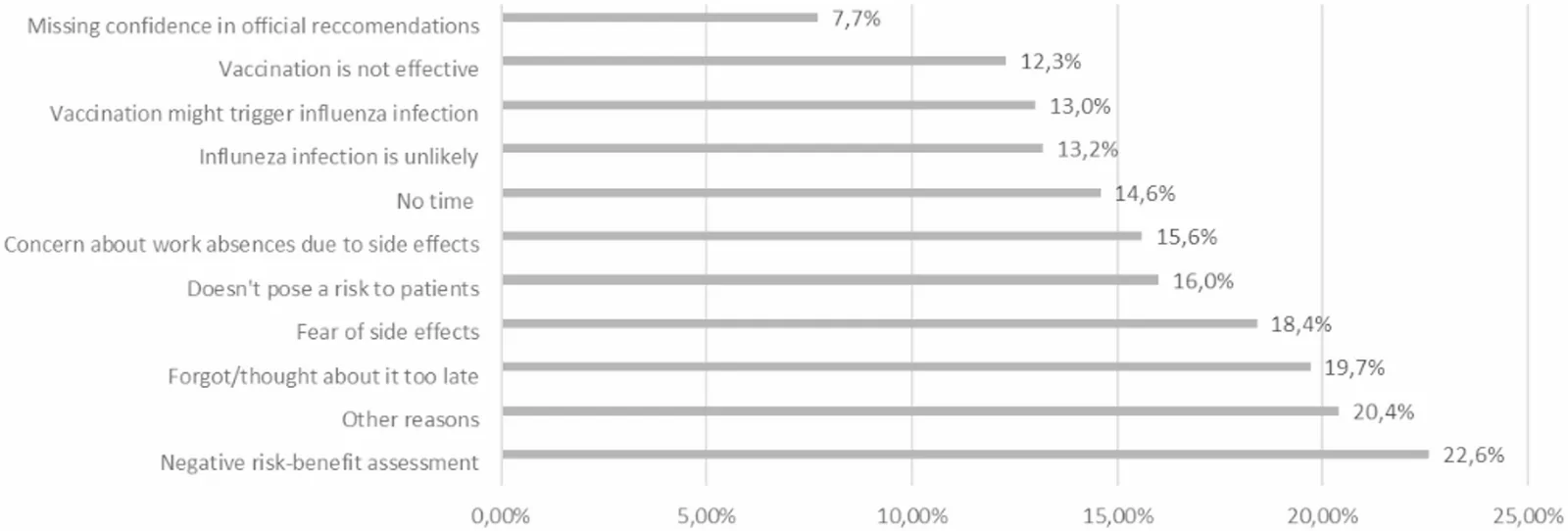
Reasons for refusing the influenza vaccination among German HCWs

Among German HCWs, the main barriers to receive vaccinations against seasonal influenza could be assigned to the CFIR domain “characteristic of the individual”. These were predominantly a low risk perception (negative risk-benefit assessment, no perceived risk to patients, flu seems to be unlikely), and missing prioritization of the vaccination (forgetting the vaccination or thinking about it too late, not having enough time to get vaccinated). Additionally, low confidence in the safety of influenza vaccinations (the vaccination could trigger influenza; potential absence from work due to adverse reactions and concerns about potential side effects) were cited. Figure 1 shows the reasons given by the German HCWs for not getting vaccinated against influenza (6.642 mentions out of *n* = 3.508 people).

Response categories with less than 5% of all mentions were not graphically represented. These included: statements that it was sufficient if colleagues were vaccinated, that there was no active recommendation from the occupational health service or the employer, and a general rejection of vaccinations. Practical barriers were also rarely mentioned and therefore play a minor role for hospital staff, like statements that the vaccination recommendation was unknown and dissatisfaction with the hospital’s vaccination program.

**Fig. 2:**
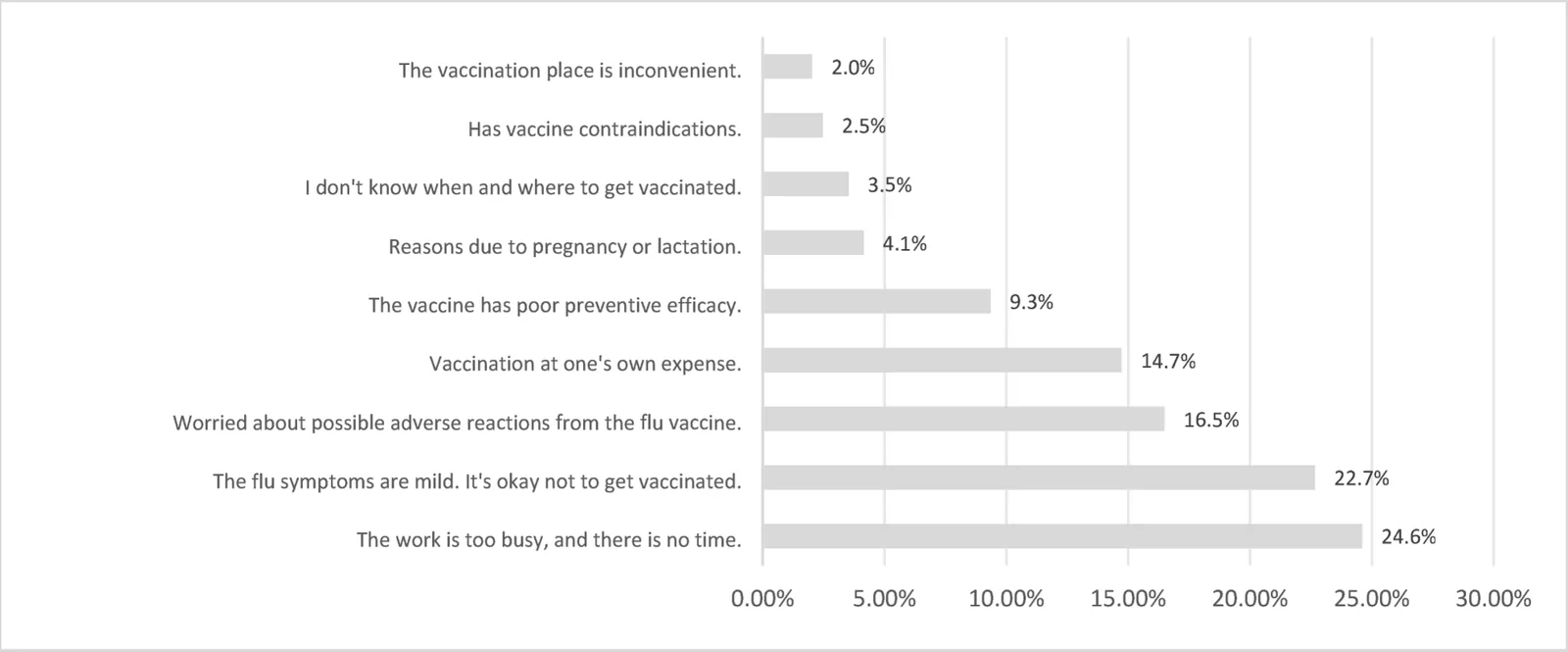
Reasons for refusing the influenza vaccination among Chinese HCWs

Among Chinese HCWs, the main barriers to receive vaccinations against seasonal influenza could be assigned to the CFIR domain “inner setting”. These were namely organizational issues and time restrictions due to high workload within their medical facilities (The work is too busy, and there is no time), followed by a low risk perception (The flu symptoms are mild, it’s okay not to get vaccinated.) as well as concerns about side effects (Worried about possible adverse reactions from the flu vaccine.). Figure 2 shows the reasons given by the Chinese HCWs for refusing to get vaccinated against influenza (1.134 mentions out of *n* = 378 people). Altogether, *n* = 503 (73.0%) of the Chinese HCWs were willing to recommend the vaccination against seasonal influneza to their patients.

### Psychological determinants of vaccination readiness

When we asked for the psychological determinants of vaccination readiness, German as well as Chinese HCWs showed very high levels of confidence into the safety and effectiveness of the influenza vaccine (confidence; German HCWs mean 3.9 vs. Chinese HCWs mean 4.4). Another driver for the seasonal vaccination against seasonal influenza was collective responsibility (German HCWs mean 3.8 vs. Chinese HCWs mean 4.5). In contrast to German HCWs, Chinese HCWs showed an higher level of calculation (extensive information searching and evaluation regarding vaccinations; German HCWs mean 3.5 vs. Chinese HCWs mean 4.4).

**Fig. 3:**
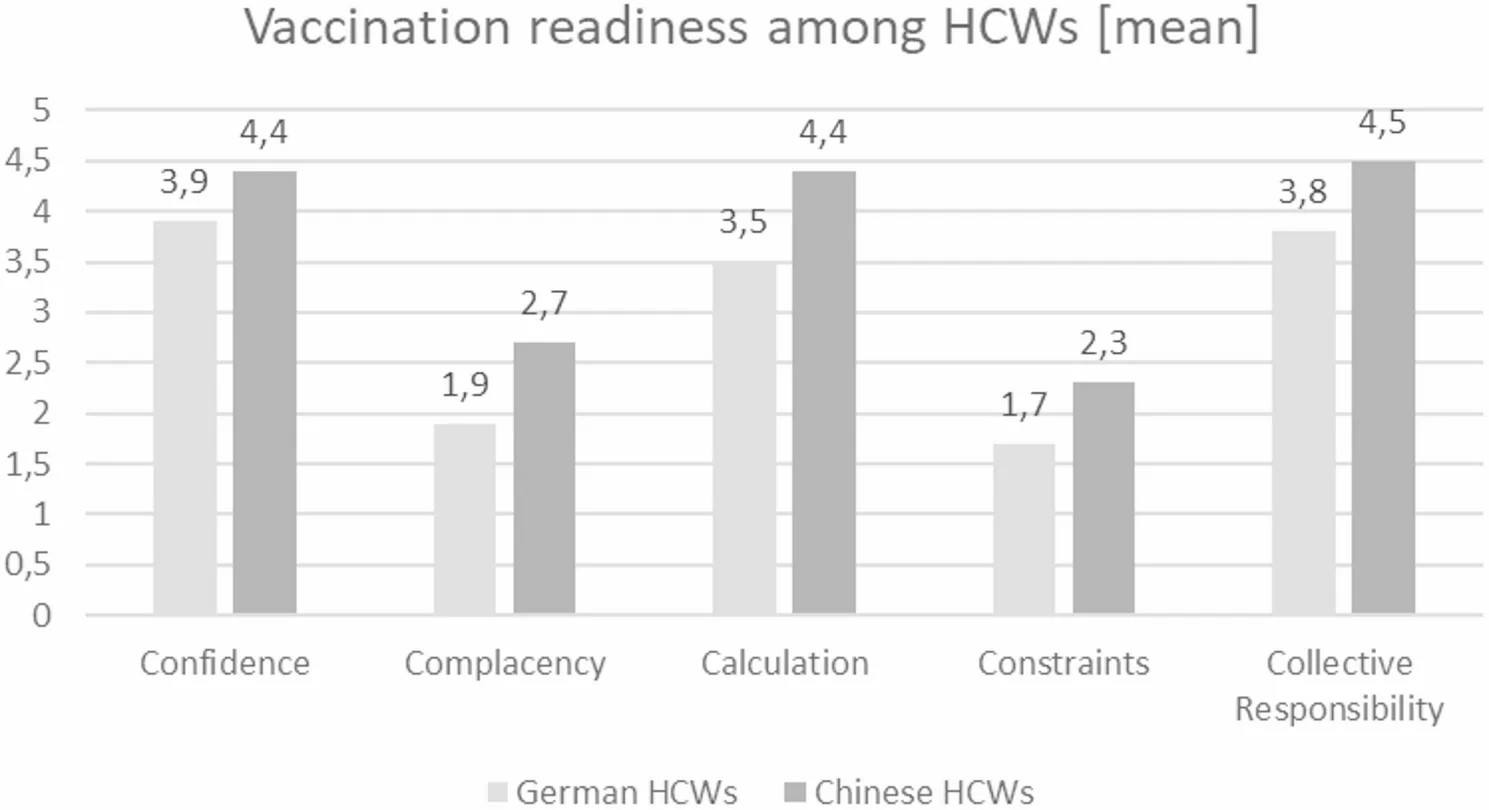
Vaccination readiness among HCWs

Figure 3 shows the degree to which HCWs agreed with the statements of the 5 C model on a scale from 1 = strongly disagree to 5 = strongly agree.

## Discussion

### Summary of findings

HCWs influence influenza vaccine uptake as both providers and recipients. Their decisions are shaped by confidence and collective responsibility, as well as different implementation factors. Nurses and physicians show contrasting uptake patterns in Germany and China, influenced by organizational barriers within the medical facilities.

### Characteristics of the individual

HCWs serve as both providers, role models and recipients of vaccination. Our results show, that HCWs tend to get vaccinated against seasonal influenza more likely with increasing age, which is line with existing literature [4]. Vaccinated HCWs are more likely to recommend influenza vaccinations to their patients [5] and play a vital role in increasing influenza vaccine uptake among other high-risk populations [18]. This study identified vaccine confidence as well as collective responsibilty as main drivers for vaccination reasdiness, supporting exisiting evidence [19]. Similar to previous researches, concerns about vaccine safety and effectiveness remain major reasons that affect their vaccination decision [20–22].

Calculation refers to individuals’ engagement in extensive information searching and evaluation regarding vaccinations to derive a good decision [8]. People with high calculation scores are more exposed to ambivalent or negative information and weigh potential risks more heavily, resulting in reduced vaccination readiness and actual vaccination behavior [8, 23].

The influence of medical training on calculation, appears ambivalent. While medical expertise improves the quality of risk assessment, studies show that medically trained individuals often make vaccination decisions less based on calculation and more on guidelines and experience [23, 24]. This suggests that professional expertise partially replaces explicit benefit-risk assessments with institutional trust. Overall, these findings suggest that calculation should be understood less as an indicator of informed decision-making and more as an expression of a specific decision-making style, which might be moderated by health literacy, confidence, and contextual factors.

### Inner setting

Heavy workload and lack of time are major barriers preventing HCWs from receiving influenza vaccination [18, 25], even if seasonal vaccinations are offered at work places. While primary care facilities provide vaccination services, their schedules primarily target the general public and overlap with HCWs’ working hours. Measures such as priority lanes or extended service hours are limited. Future efforts should focus accessability of vaccination services for HCWs as they are more likely to recommend the vaccination to their patients, if they have received the vaccinations themselves [26]. Although mandatory vaccination policies are effective, they remain highly controversial, primarily due to concerns about HCWs’ autonomy [22].

### Outer setting

The comparatively higher vaccination rates among the German study participants could partly be explained by cost coverage. All vaccinations recommended by the Standing Committee on Vaccination in Germany are fully covered by the respective health insurance [27]. In China, influenza vaccination is self-paid and not part of the National Immunization Program (NIP). Although national policies recommend prioritizing HCWs and reducing the cost for high-risk groups [28], practical implementation varies across regions due to dependence on local government funding support [29].

### Process of implementation

Our study reveals differences in influenza vaccination rates among different types of HCWs, and these patterns differ markedly between Germany and China. In Germany, nurses show lower vaccination rates than physicians. In contrast, data from China indicate the opposite trend, supporting international evidence [19, 30–32]. Regarding medical education, the pathway for training physicians is similar between China and Germany. Both require systematic course learning and vocational training [33]. However, substantial differences exist in the training of nurses [34, 35]. In Germany, nursing education follows a dual vocational training model with a strong emphasis on practical skills. While Chinese nurses typically complete college or university-level medical training that includes coursework in infectious diseases, immunology, and public health, and they also receive extensive continuing medical education after entering the workforce.

Differences in the healthcare system may also contribute to these contrasting patterns. In Germany, vaccination services are primarily provided by general practitioners [36]. In China’s primary healthcare system, however, nurses and public health practitioners serve as the main vaccination providers [31]. A qualitative study from Shandong Province further indicated that some general practitioners do not even consider vaccination services part of their professional responsibilities [26].

### Characteristics of the intervention

Existing studies have proposed several types of interventions to promote influenza vaccination among HCWs. These strategies can generally be categorized into three types: policy measures, educational and promotional strategies, and organizational interventions. Li et al. found that policies offering free influenza vaccination to HCWs are an important measure to increase both vaccination willingness and uptake [37]. Previous research also discussed the use of mandatory vaccination policies, though such approaches require careful consideration of ethical and social implications [38]. Educational and promotional strategies aim to improve HCWs’ knowledge of influenza and its vaccines, correct misconceptions, and enhance vaccination willingness. A systematic review reported that the key intervention education and promotion increased vaccination rate relatively by 65.9% [39]. The intervention strategies include lectures/training, counseling, distribution of educational materials, regular reminders, and examples from key personnel in the department [40–44]. Organizational interventions refer to supportive measures implemented on the organizational level of the medical facility improve convenience and accessibility. Evidence suggests that extending operating hours of vaccination clinics, establishing convenient/occupational vaccination sites, streamlining vaccination procedures, and introducing targeted incentives can significantly increase uptake among HCWs [45, 46].

Given the geographical variations in the psychological and contextual determinants of vaccination readiness, it is reasonable to assume that a combination of institutional recommendations, simplified access to vaccination during working hours, and a policy of free vaccination could lead to a significant increase in vaccination rates among medical personnel in China—particularly given that China relies heavily on a system of community solidarity, yet individuals are required to bear the cost of vaccination themselves [21]. From a German perspective, improved information regarding risks and benefits—based on the “Health Belief Model”—as well as reliable reminder systems could be of crucial importance [47, 48]. This can be explained, among other things, by a strongly individualistic attitude—one that should, take a back seat when it comes to vaccination as a public health measure. However, reviews also emphasize that combined strategies are more effective than single interventions [22].

### Strengths and limitations

This comparative analysis, with its large number of study participants in two very different health systems, allows a good understanding of potenial interventions and implementation factors that might influence vaccination readiness in HCWs.

Several limitations should be considered when interpreting these findings. First, the two surveys were not conducted during the same time period, and it was not possible to achieve the same group sizes for a balanced comparison. The use of a convenience sample limits the generalizability of the results to other healthcare settings and populations. All variables, including vaccination status and attitudes, were self-reported, which may introduce recall or social desirability bias. The validated questionnaires to assess vaccination readiness in German and Chinese differ both in their specific wording and in their response scales, thus limiting comparability. From a methodological standpoint, there was sparse data in the lower range of the 5 C confidence domain, potentially limiting the ability to detect associations at lower confidence levels. Additionally, the number of participants who reported not having a workplace vaccine offer was small, resulting in low cell counts and less precise estimates for this subgroup.

## Conclusions

Although HCWs are a high-risk group for infectious diseases with high levels of vaccination readiness, the overall vaccination rate remains low. Vaccination readinss among German and Chinese HCWs is shaped by confidence, collective responsibility, and context specific implementation factors. Effective interventions might include free vaccination policies, intensified educational training, and reduced organizational barriers.

## Data Availability

The datasets used and/or analysed during the current study are available from the corresponding author on reasonable request.

## Abbreviations

CDC: Centers for Disease Control
CFIR: Consolidated Framework for Implementation Research
HCWs: Healthcare workers
RKI: Robert Koch-Institute

## References

[1] Iuliano AD, Roguski KM, Chang HH, Muscatello DJ, Palekar R, Tempia S, et al. Estimates of global seasonal influenza-associated respiratory mortality: a modelling study. Lancet. 2018;391(10127):1285–300. 29248255 10.1016/S0140-6736(17)33293-2 PMC5935243

[2] Hui Z, Jian-Shi H, Xiong H, Peng L, Da-Long Q. An analysis of the current status of hospital emergency preparedness for infectious disease outbreaks in Beijing, China. Am J Infect Control. 2007;35(1):62–7. 17276793 10.1016/j.ajic.2006.03.014 PMC7132672

[3] Shattock AJ, Johnson HC, Sim SY, Carter A, Lambach P, Hutubessy RCW, et al. Contribution of vaccination to improved survival and health: modelling 50 years of the Expanded Programme on Immunization. Lancet. 2024;403(10441):2307–16. 38705159 10.1016/S0140-6736(24)00850-X PMC11140691

[4] Dardas LA, Al-Leimon O, Jaber AR, Saadeh M, Al-Leimon A, Al-Hurani A, et al. Flu shots unveiled: A global systematic review of healthcare providers’ uptake of, perceptions, and attitudes toward influenza vaccination. Vaccines. 2023;11(12):1760. 38140165 10.3390/vaccines11121760 PMC10747442

[5] Fan J, Zhang R, Zhang Q, Cong S, Song Y, Schluter WW, et al. Healthcare workers from two sites in China in 2018–2019 unlikely to receive and recommend influenza vaccination: A qualitative study following a quantitative analysis to improve future interventions. Influenza Other Respir Viruses. 2025;19(11):e70157. 41177808 10.1111/irv.70157 PMC12580223

[6] Neufeind J, Wenchel R, Boedeker B, Wicker S, Wichmann O. Monitoring influenza vaccination coverage and acceptance among health-care workers in German hospitals – results from three seasons. Hum Vaccines Immunotherapeutics. 2021;17(3):664–72. 10.1080/21645515.2020.1801072 PMC7993141 33124954

[7] Schmid P, Rauber D, Betsch C, Lidolt G, Denker M-L. Barriers of influenza vaccination intention and behavior–a systematic review of influenza vaccine hesitancy, 2005–2016. PLoS ONE. 2017;12(1):e0170550. 28125629 10.1371/journal.pone.0170550 PMC5268454

[8] Betsch C, Schmid P, Heinemeier D, Korn L, Holtmann C, Böhm R. Beyond confidence: Development of a measure assessing the 5 C psychological antecedents of vaccination. PLoS ONE. 2018;13(12):e0208601. 30532274 10.1371/journal.pone.0208601 PMC6285469

[9] Kaddour O, Ben Mabrouk A, Arfa S, Lassoued N, Berriche O, Chelli J. Knowledge and attitudes of healthcare workers about influenza vaccination. Infect Disease Health. 2024;29(4):203–11. 38679564 10.1016/j.idh.2024.04.005

[10] Yu M, Yao X, Liu G, Wu J, Lv M, Pang Y, et al. Barriers and facilitators to uptake and promotion of influenza vaccination among health care workers in the community in Beijing, China: A qualitative study. Vaccine. 2022;40(14):2202–8. 35248421 10.1016/j.vaccine.2022.02.060

[11] Arueyingho Victoria O. Comparative Health Systems: Germany Versus China. Int J Med Reviews. 2021;8(4):170–83.

[12] Akintola A, Newbury-Birch D, Kilinc S. Bridging the gap between research evidence and its implementation in public health practice: case studies of embedded research model. BMC Public Health. 2024;24(1):1299. 38741039 10.1186/s12889-024-18727-z PMC11092060

[13] Atkins L, Francis J, Islam R, O’Connor D, Patey A, Ivers N, et al. A guide to using the Theoretical Domains Framework of behaviour change to investigate implementation problems. Implement Sci. 2017;12(1):77. 28637486 10.1186/s13012-017-0605-9 PMC5480145

[14] Damschroder LJ, Aron DC, Keith RE, Kirsh SR, Alexander JA, Lowery JC. Fostering implementation of health services research findings into practice: a consolidated framework for advancing implementation science. Implement Sci. 2009;4:50. 19664226 10.1186/1748-5908-4-50 PMC2736161

[15] Zhang E, Dai Z, Wang C, Hu J, Wang S, Zhang L, et al. Targeting COVID-19 vaccine hesitancy among nurses in Shanghai: A latent profile analysis. Front Public Health. 2022;10:953850. 36187664 10.3389/fpubh.2022.953850 PMC9515966

[16] Yang L, Zhen S, Li L, Wang Q, Yang G, Cui T, et al. Assessing vaccine literacy and exploring its association with vaccine hesitancy: A validation of the vaccine literacy scale in China. J Affect Disord. 2023;330:275–82. 36907456 10.1016/j.jad.2023.03.014

[17] Damschroder LJ, Reardon CM, Widerquist MAO, Lowery J. The updated Consolidated Framework for Implementation Research based on user feedback. Implement Sci. 2022;17(1):75. 36309746 10.1186/s13012-022-01245-0 PMC9617234

[18] Yang L, Chen J, Li Y, Chen X. Willingness to receive influenza vaccines among medical staff in China: A meta-analysis. J Prev Med. 2024;36(2):109-14. 10.19485/j.cnki.issn2096-5087.2024.02.005.

[19] Sallam M, Ghazy RM, Al-Salahat K, Al-Mahzoum K, AlHadidi NM, Eid H, et al. The Role of Psychological Factors and Vaccine Conspiracy Beliefs in Influenza Vaccine Hesitancy and Uptake among Jordanian Healthcare Workers during the COVID-19 Pandemic. Vaccines. 2022;10(8):1355. 36016243 10.3390/vaccines10081355 PMC9413675

[20] Lytras T, Kopsachilis F, Mouratidou E, Papamichail D, Bonovas S. Interventions to increase seasonal influenza vaccine coverage in healthcare workers: A systematic review and meta-regression analysis. Hum Vaccines Immunotherapeutics. 2016;12(3):671–81. 10.1080/21645515.2015.1106656 PMC4964628 26619125

[21] Yi H, Yang Y, Zhang L, Zhang M, Wang Q, Zhang T, et al. Improved influenza vaccination coverage among health-care workers: Evidence from a web-based survey in China, 2019/2020 season. Hum Vaccines Immunotherapeutics. 2021;17(7):2185–9. 10.1080/21645515.2020.1859317 PMC8189132 33497309

[22] Dini G, Toletone A, Sticchi L, Orsi A, Bragazzi NL, Durando P. Influenza vaccination in healthcare workers: A comprehensive critical appraisal of the literature. Hum Vaccines Immunotherapeutics. 2018;14(3):772–89. 10.1080/21645515.2017.1348442 PMC5861785 28787234

[23] Dubé E, Laberge C, Guay M, Bramadat P, Roy R, Bettinger J. Vaccine hesitancy: an overview. Hum Vaccin Immunother. 2013;9(8):1763–73. 23584253 10.4161/hv.24657 PMC3906279

[24] Dubé E, Gagnon D, Nickels E, Jeram S, Schuster M. Mapping vaccine hesitancy–country-specific characteristics of a global phenomenon. Vaccine. 2014;32(49):6649–54. 25280436 10.1016/j.vaccine.2014.09.039 PMC5355208

[25] Liu H, Tan Y, Zhang M, Peng Z, Zheng J, Qin Y, et al. An internet-based survey of influenza vaccination coverage in healthcare workers in China, 2018/2019 season. Vaccines. 2019;8(1):6. 31887994 10.3390/vaccines8010006 PMC7158694

[26] Liu Y, Liu T, Yao M, Kou Z, Li R. Exploring barriers to influenza vaccine uptake and recommendation among healthcare providers in the community in China: A qualitative study. Hum Vaccines Immunotherapeutics. 2024;20(1):2352916. 10.1080/21645515.2024.2352916 PMC11095569 38744298

[27] Paul KT, Loer K. Contemporary vaccination policy in the European Union: tensions and dilemmas. J Public Health Policy. 2019;40(2):166–79. 30894672 10.1057/s41271-019-00163-8

[28] Twg IV, National Immunization Advisory C, Technical Working G. Technical guidelines for seasonal influenza vaccination in China (2023–2024). Zhonghua liu xing bing xue za zhi=. Zhonghua liuxingbingxue zazhi. 2023;44(10):1507–30. 37875437 10.3760/cma.j.cn112338-20230908-00139

[29] Kong QF, Zhang X, Tang L, Hou Q, Zhang GM, Hao LX, et al. Influenza vaccine coverage among healthcare workers in the 2019 season, their willingness to receive influenza vaccine in the 2020 season, and factors influencing coverage and willingness. Chin J Vaccines Immun. 2021;27:311–6.

[30] Ding Y, Xu J, Ying W, Lu Y, Xu H, Zhang A, et al. Coverage and influencing factors of influenza vaccination among community healthcare workers in shanghai, 2023–2024. Hum Vaccines Immunotherapeutics. 2025;21(1):2588025. 10.1080/21645515.2025.2588025 PMC12645848 41267334

[31] Li M, Sun B, Su JF. Status, recommendation behavior and influencing factors of influenza vaccination among primary health care workers: A cross-section survey. Chin J Health Educ. 2021;37(12):1095–100.

[32] Schug C, Erim Y, Geiser F, Hiebel N, Beschoner P, Jerg-Bretzke L, et al. [Vaccination willingness against COVID-19 among healthcare workers in Germany: Results from a University Medicine Network survey between November 2020 and January 2021]. Bundesgesundheitsblatt Gesundheitsforschung Gesundheitsschutz. 2022;65(1):74–85. 34554277 10.1007/s00103-021-03418-6 PMC8458789

[33] Xu Y, Hao Y, Chen T, Li X. Structural and policy overview of medical education in Germany. Glob Med Educ. 2025;2(1):25-37. 10.1515/gme-2024-0016.

[34] Wang X. Status quo of German vocational education nursing specialty and its enlightenment to reform of resident teaching. Chin Nurs Res. 2018;32(20):3301-03.

[35] Zhen R. Enlightenment of the German dual system educationconcept on the innovative and development of 3 + 2’college students for nursing education. Hainan Med J. 2024;35(6):876–80.

[36] Wilkinson E, Jetty A, Petterson S, Jabbarpour Y, Westfall JM. Primary Care’s Historic Role in Vaccination and Potential Role in COVID-19 Immunization Programs. Ann Fam Med. 2021;19(4):351–5. 33707190 10.1370/afm.2679 PMC8282305

[37] Li QQ, Yang Y, Ma HZ. Evaluation on effect of free influenza vaccine among medical staff and suggestions for its improvement. Practical Prev Med. 2021;28(9):1111–3.

[38] Ghandora H, Halperin DM, Isenor JE, Taylor BA, Fullsack P, Di Castri AM, et al. Knowledge, attitudes, behaviours, and beliefs of healthcare provider students regarding mandatory influenza vaccination. Hum Vaccines Immunotherapeutics. 2019;15(3):700–9. 10.1080/21645515.2018.1543523 PMC6605728 30395762

[39] Schumacher S, Salmanton-García J, Cornely OA, Mellinghoff SC. Increasing influenza vaccination coverage in healthcare workers: a review on campaign strategies and their effect. Infection. 2021;49(3):387–99. 33284427 10.1007/s15010-020-01555-9 PMC7720031

[40] Abramson ZH, Avni O, Levi O, Miskin IN. Randomized trial of a program to increase staff influenza vaccination in primary care clinics. Annals Family Med. 2010;8(4):293–8. 10.1370/afm.1132 PMC2906523 20644183

[41] Borgey F, Henry L, Lebeltel J, Lescure P, Le Coutour X, Vabret A, et al. Effectiveness of an intervention campaign on influenza vaccination of professionals in nursing homes: A cluster-randomized controlled trial. Vaccine. 2019;37(10):1260–5. 30738645 10.1016/j.vaccine.2019.01.066

[42] Costantino C, Restivo V, Gaglio V, Lanza GLM, Marotta C, Maida CM, et al. Effectiveness of an educational intervention on seasonal influenza vaccination campaign adherence among healthcare workers of the palermo university hospital, italy. Ann Ig. 2019;31(1):35–44. 30554237 10.7416/ai.2019.2256

[43] Jung Y, Kwon M, Song J. Stepwise intervention including 1-on-1 counseling is highly effective in increasing influenza vaccination among health care workers. Am J Infect Control. 2017;45(6):635–41. 28063732 10.1016/j.ajic.2016.11.012

[44] Llupià A, García-Basteiro AL, Olivé V, Costas L, Ríos J, Quesada S, et al. New interventions to increase influenza vaccination rates in health care workers. Am J Infect Control. 2010;38(6):476–81. 20421140 10.1016/j.ajic.2010.01.013

[45] Bianchi FP, Stefanizzi P, Cuscianna E, Di Lorenzo A, Martinelli A, Tafuri S. Effectiveness of on-site influenza vaccination strategy in italian healthcare workers: A systematic review and statistical analysis. Expert Rev Vaccines. 2023;22(1):17–24. 36409195 10.1080/14760584.2023.2149500

[46] Podczervinski S, Stednick Z, Helbert L, Davies J, Jagels B, Gooley T, et al. Employee influenza vaccination in a large cancer center with high baseline compliance rates: Comparison of carrot versus stick approaches. Am J Infect Control. 2015;43(3):228–33. 25728148 10.1016/j.ajic.2014.11.025 PMC4372134

[47] Zhelyazkova A, Kim S, Klein M, Prueckner S, Horster S, Kressirer P, et al. COVID-19 Vaccination Intent, Barriers and Facilitators in Healthcare Workers: Insights from a Cross-Sectional Study on 2500 Employees at LMU University Hospital in Munich, Germany. Vaccines. 2022;10(8):1231. 36016119 10.3390/vaccines10081231 PMC9412572

[48] Schelling J, Thorvaldsson I, Sanftenberg L. Digital vaccination management systems may improve immunization rates in primary healthcare. Bundesgesundheitsblatt Gesundheitsforschung Gesundheitsschutz. 2019;62(4):433–9. 30820616 10.1007/s00103-019-02912-2

